# Liquid Biopsy-Based Colorectal Cancer Screening via Surface Markers of Circulating Tumor Cells

**DOI:** 10.3390/diagnostics11112136

**Published:** 2021-11-17

**Authors:** Francis Yew Fu Tieng, Nadiah Abu, Siti Nurmi Nasir, Learn-Han Lee, Nurul-Syakima Ab Mutalib

**Affiliations:** 1 UKM Medical Molecular Biology Institute (UMBI), Universiti Kebangsaan Malaysia, Kuala Lumpur 56000, Malaysia; francistieng@yahoo.com.my (F.Y.F.T.); nadiah.abu@ppukm.ukm.edu.my (N.A.); ctnurminasir@ppukm.ukm.edu.my (S.N.N.); 2 Novel Bacteria and Drug Discovery Research Group, Microbiome and Bioresource Research Strength, Jeffrey Cheah School of Medicine and Health Sciences, Monash University of Malaysia, Subang Jaya 47500, Selangor, Malaysia; 3 Faculty of Health Sciences, Universiti Kebangsaan Malaysia, Kuala Lumpur 50300, Malaysia

**Keywords:** circulating tumor cells enrichment, cell surface markers, colorectal cancer, blood-based screening, non-invasive, multiplex

## Abstract

Colorectal cancer (CRC) is ranked second for cancer-related deaths worldwide with approximately half of the patients being diagnosed at the late stages. The untimely detection of CRC results in advancement to the metastatic stage and nearly 90% of cancer-related deaths. The early detection of CRC is crucial to decrease its overall incidence and mortality rates. The recent introduction of circulating tumor cells (CTCs) has enabled a less invasive sampling method from liquid biopsies, besides revealing key information toward CRC metastasis. The current gold standard for CTC identification is the CellSearch® system (Veridex). This first-generation instrumentation relies on a single cell surface marker (CSM) to capture and count CTCs. Detection of CTCs allows the identification of patients at risk for metastasis, whereas CTC enumeration could improve risk assessment, monitoring of systemic therapy, and detection of therapy resistance in advanced metastatic CRC. In this review, we compared the pros and cons between single CSM-based CTC enrichment techniques and multi-marker-based systems. We also highlighted the challenges faced in the routine implementation of CSM-dependent CTC detection methods in CRC screening, prediction, prognosis, disease monitoring, and therapy selection toward precision medicine, as well as the dwelling on post-CTC analysis and characterization methods.

## 1. Introduction

Colorectal cancer (CRC) is among the most common global health issues with a startling surge in incidence (10.2%) and mortality (9.2%) rates [1]. The time of diagnosis directly corresponds to the overall survival of CRC patients. Localized cancer lesions could be easily removed during its early stages (5 year survival rate > 90%) [2,3]. However, when CRC patients advance into the late/metastatic stage, only half of them survive within five years [4,5]. There is no effective treatment for patients diagnosed with metastasis or stage IV nonresectable tumors, and palliative therapies are often given only to relieve, delay, or prevent symptoms [6].

Currently, the gold standard for CRC diagnosis includes both colonoscopy [7] and histopathological examinations, where the two of them complement each other [8]. The former is the most sensitive procedure for CRC diagnosis as it permits the visualization and removal/surgery of colorectal tumors and pre-cancerous lesions (adenomas and polyps) [9,10]. When coupled with the latter, analysis of the excised tumors is possible, classifying them into different tumor stages/groups based on their clinicopathological features for better treatment selections [11]. For instance, the American Joint Committee on Cancer (AJCC) staging manual classifies CRC patients into five different stages (stage 0 to IV) based on the presence of tumors (T), the number of lymph node metastases (N), and the presence of distant metastases (M) [12,13]. However, these procedures are compromised and limited among patients due to their invasive nature, high cost, unstandardized protocols, expertise and apparatus requirements, labor-intensiveness, the low number of advanced neoplasms at specific sites and nonrepeatability over time, besides difficulties in predicting the extent of metastasis, distant metastases, and cancer heterogeneity accurately [14,15,16,17,18]. In short, the primary cause of cancer mortality is metastasis. Therefore, there is a need for a simpler, less invasive detection method to provide clear scientific evidence and improve early detection among CRC patients [19,20]. 

## 2. Circulating Tumor Cells Shed Insights toward Liquid Biopsy-based CRC Screening

**Figure 1:**
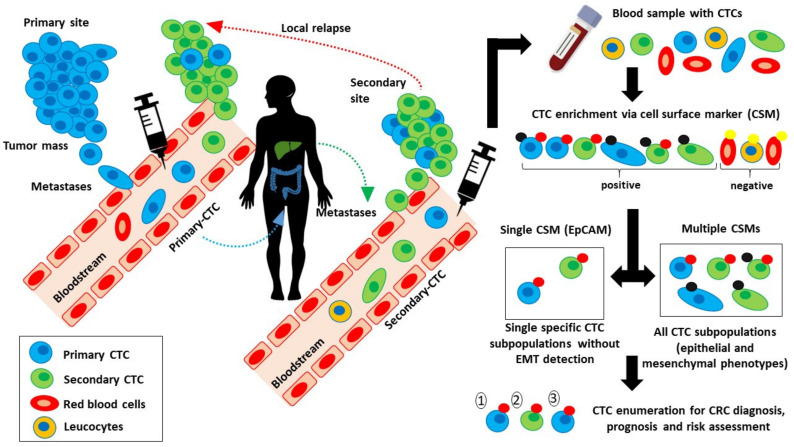
Sources of CTCs and the comparison between single-based and multi-marker-based CSM-dependent CTC enrichment.

Circulating tumor cells (CTCs) denote epithelial cancer cells found in the bloodstream [21,22,23]. Their detection differs drastically from person to person but is often observed in patients with metastatic CRC (mCRC) [24,25,26,27]. Lately, CTCs have been intensively discussed due to the noninvasive identification technique, which enables the extraction of adequate diagnostic and predictive information directly from blood as compared to the invasive conventional biopsy [28]. In addition, CTCs have the potential to provide direct access to all phases of CRC carcinogenesis and are the main vehicles for metastatic reoccurrence [29,30]. During metastasis, tumor cells (1 million cells per gram of tumor tissue) escape from the tumor mass into the bloodstream, forming CTCs [22,31]. Although the majority of the CTCs undergo cell detachment-induced apoptosis, a small portion of the cells survive and form a micrometastasis [32,33,34]. From there, only a few of them progress to form macroscopic tumors [35,36]. These cells aggregate and form secondary tumor sites, which then release CTCs back into the bloodstream and might attach to the initial primary site (local relapse), as illustrated in Figure 1 [37,38]. In short, CTCs are believed to generate valuable information and provide critical insights into the aggressive nature of the tumor and also a better understanding of the underlying biology related to CRC dissemination and metastasis [21,39]. Thus, the application of CTCs as cancer biomarkers offers a more effective alternative to detect, analyze, treat, and monitor CRC therapeutic responses and disease progression [40,41].

## 3. Existing Blood-based Biomarkers Are Not Effective with Low Accuracy

Previous studies had proven the implementation of biomarkers from blood circulation as a noninvasive method for CRC screening, particularly during its early stages (stage I or premalignant stage) [42,43,44,45,46,47,48,49]. Despite the discovery of innumerable blood-based CRC-specific markers, follow-up cohort studies including a large population of patients are lacking, and relatively few of them could be translated into clinical practice [50,51,52,53,54]. 

To date, the two most recognized CRC-specific antigens are carcinoembryonic antigen (CEA) and carbohydrate antigen (CA 19.9) [55,56,57,58,59]. However, both of these biomarkers are not effective in CRC detection, due to the overlapping/close proximity of ranges of concentration across different stages [60] and the fact that only certain CRC patients have expressed elevations in CEA (43%) and CA19.9 (27%) in blood serum, hindering accurate distinction [61]. Other factors include the nonspecificity of CEA and CA 19.9 toward a particular histological type or origin of the carcinoma, false-positive results from the elevation of CEA levels due to benign conditions (e.g. hepatitis, pancreatitis, obstructive pulmonary disease, and inflammatory bowel disease), and analytical variables such as variations in sampling and storage methods, patients’ condition, and stability of the biomarkers [62,63]. Thus, the identification of a rapid, sensitive, and CRC marker-specific method is crucial in developing accurate assays for effective CRC detection from peripheral blood [64]. 

## 4. “Gold Standard”: Single CTC-Specific Cell Surface Marker-Positive Enrichment

The pioneer stage in applying CTCs as cancer biomarkers is the ability to capture and detect CTCs from blood samples [65]. The detection of CTCs is challenging majorly because of its rarity (1 CTC per 10^7^ to 10^9^ hematological cells/mL), the short half-life of a few hours ex vivo, the lack of a single ubiquitous/universal CTC-specific marker, and technical limitations such as low separation efficiency and low recovery rates [66,67,68,69,70,71]. This has led to the invention of isolation devices that focus on exploiting cell surface markers (CSMs)/antigens expressed on CTCs but not expressed on the surrounding nontarget cells (e.g., leucocytes and red blood cells). Some examples of the CTC enrichment techniques are immunoaffinity-based purification (biological), and biophysical isolation methods that rely on the differential size and/or density of CTCs and di-electrophoretic-based strategies [72,73]. Among them, CTC enrichment by immunoaffinity is the most widely used strategy for CTC isolation. 

Immunoaffinity-based CTC purification is categorized into two main groups, namely positive and negative enrichment strategies. Positive enrichment isolates CTCs by targeting specific CSMs, whereas the latter captures background/nontarget cells by targeting CSMs deficient in CTCs. Currently, the most well-known and established positive enrichment method is the CellSearch® system (Veridex). This Food and Drug Administration (FDA)-approved first-generation instrumentation relied on a single CSM epithelial cell adhesion molecule (EpCAM) to capture CTCs, followed by CTC enumeration to provide cancer prediction, prognosis, and clinical outcomes [74,75,76,77,78]. The detection of CTCs enabled the identification of patients at risk for metastasis originating from localized CRC, whereas CTC enumeration could improve risk assessment, monitoring of systemic therapy, and detection of therapy resistance in advanced mCRC. Since its approval, enrichment techniques based on a single specific CSM have become the gold standard for CTC isolation [79,80]. To summarize, the primary principle of this method includes both targeting the antigen expression of CTCs (detection) and counting of CTC (enumeration).

Despite the advancement in monoclonal antibodies, microfluidics, fluorescence, and laser technologies, EpCAM remains the principal CSM for most of the CTCs enrichment methods available for CRC [81]. In 2019, Gupta and the coworkers evaluated the assay specificity and clinical feasibility of the CellMax CTC detection assay (CellMax Life) in a cohort study. This single specific EpCAM-dependent assay that is based on microfluidic chip technology could accurately enrich CTC from peripheral blood with a high sensitivity (80%), specificity (80%), and recovery rate up to 80.8% when spiked with HT29 cells [82]. In the same year, Tsai et al. verified the single EpCAM-dependent CellMax platform as an early cancer detection method due to its ability to relate the captured epithelial CTCs count to different CRC stages (adenomas, stage I, II, III, IV) with a positive detection rate up to 94.5% (307/325 patients) [30]. Following this, a polymeric chip coated with solely EpCAM was developed by Kure and the coauthors to enrich CTCs from CRC patients. This protocol not only showed a significantly higher positive detection rate than the CA19.9 test but also validated CTCs as effective markers for stage II and III CRC, who often exhibit negative conventional serum marker test results [83]. In 2020, a group of Australian researchers applied the single EpCAM-based magnetic CTC isolation technique known as IsoFlux (Fluxion Biosciences) in a comparative, longitudinal study. Interestingly, they discovered that CTCs with high microsatellite instability were associated with a rise in CTCs released intra-operatively and post-operatively [84]. In a nutshell, the single specific CSM-based CTC enrichment technique is capable of CRC early screening, prognosis, and prediction of treatment, as well as disease progression/treatment effects monitoring.

## 5. Single Specific CSM-based CTC Enrichment Strategy Had Its Limitations

Although targeting EpCAM on CTCs from CRC (epithelial origin) seems to be the best option to distinguish between CTCs and normal blood cells with mesenchymal phenotypes, the overreliance on a single specific CSM resulted in a selection bias [79], where only CTCs that predominantly retain epithelial characteristics (high EpCAM levels) are enriched, excluding a subpopulation of CTCs with mesenchymal traits (low or no EpCAM expressed) [85,86,87]. This could have serious implications as CTCs are characterized by phenotypic plasticity that mainly reflects an epithelial-to-mesenchymal transition state (EMT), especially when progressing into mCRC and/or acquiring chemoresistance [88,89,90,91,92,93,94]. For instance, scientists from China had found out that only mesenchymal and epithelial–mesenchymal CTCs, not epithelial CTCs, were correlated with clinical stage and metastasis in CRC [95]. Moreover, increased analytic sensitivity and specificity by including more CSM markers for secondary CTC identification after the initial single EpCAM-positive enrichment did not change the fact that only EpCAM-positive CTCs were isolated. Thus, it was unsurprising that a prospective and investigator-blinded side-by-side comparison of CellSearch (pan-CK) and GILUPI CellCollector (EpCAM and pan-CK double staining) did not show significance in either the total number or the frequency of CTCs detected in both metastatic and nonmetastatic CRC patients [96]. In this context, the heterogeneity of CTCs creates a significant loss of certain CTC subpopulations, which leads to uncertainty in the accuracy of a single CSM-dependent positive analysis to identify a patient’s CTC status. 

To overcome this, negative enrichment was introduced. It captures nontarget cells (e.g., hematogenous cells), followed by the isolation of CTCs. Unlike positive enrichments, negative enrichments could harvest all types of CTCs as they are not dependent on the CSM profiles and are more competent for the discovery of cellular and transcriptomic cancer biomarkers of cancer and downstream analyses such as genetic assays, CTC culture, and xenografts [97]. Regardless of several negative methods developed such as the CellSearch® system (Veridex) [98], Cyttel method [99], RosetteSep™ system [100], subtraction enrichment, and immunostaining-fluorescence in situ hybridization (SE-iFISH) (Cytelligen) [101] and EasySep™ (StemCell Technologies) [102], all of them use lymphocyte common antigen (CD45) as the main marker to remove hematogenous cells. Similar to traditional negative enrichment, these systems employ the single specific CD45 CSM to deplete nontarget cells and elute CTCs, followed by a CTC-specific antibody cocktail (EpCAM, CK, CK3, CK18, CK19, MUC1, CD44, CD133, ALDH1, and/or CEP8) to identify the captured cells. The downside of the single CSM negative isolation is that it had less purity (ability to detect CTCs in the presence of contaminating background cells) and lower specificity (significant loss of CTCs) than positive enrichment. 

## 6. Alternative CSMs and Multiplexing Show Potential in Targeting a Wider CTC Population

To further expand the detection limit of single analyte-dependent enrichment (to include more CTC subpopulations during separation), several attempts had been conducted using different CSMs, including KRAS (Kirsten rat sarcoma viral oncogene), pan-cytokeratin (pan-CK), vimentin (VIM), cluster of differentiation (CD2, CD16, CD19, CD36, CD38, CD45, and CD66b), and/or glycophorin A. For instance, Feng and the researchers proposed the use of a lipid magnetic ball coated with KRAS to isolate CTCs from CRC with KRAS mutations. The reason for opting for KRAS over EpCAM was that (1) KRAS was closely related to CRC signaling pathways such as MAPK, PI3K, Wnt, and EGFR [103,104,105,106]; (2) almost half of the CRC patients were characterized by a mutation in the codons 12 and 13 in exon 2 of the KRAS gene [107,108]; and (3) those without the KRAS mutation tend to develop secondary KRAS mutations (~30%) during courses of targeted therapy [109,110]. Based on their results, KRAS-modified enrichment could effectively improve the capture ability of CTCs with KRAS mutation up to 92.9%, and the result was in concordance with clinical diagnosis and pathology. Their results showed that KRAS immune lipid magnetic balls could be used in the diagnosis and treatment of KRAS CRC [111].

In tackling the challenges of single CSM-dependent CTC enrichment in addressing the totality of CTCs, a multi-marker-based system could potentially isolate CTCs of different origins by covering epithelial, mesenchymal, and stem cell markers. In 2017, Soler et al. modified the RosetteSep™ System (StemCell Technologies) to include a list of CSMs (CD2, CD16, CD19, CD36, CD38, CD45, CD66b, and glycophorin A) as tetrameric antibody complexes to crosslink unwanted cells for CTC elution. CTCs were then purified via density gradient centrifugation, followed by an EPISPOT assay where specific secreted proteins were captured by an antibody-coated membrane. These immunospots were counted (one immunospot corresponded to the protein fingerprint of one viable cell). Their experiment described negative enrichment multiplexing to be capable of harvesting all types of CTCs, detecting viable CTCs at the single-cell resolution and providing downstream analysis for CTC phenotypic and protein characterization [100].

Following this, a group of scientists from France isolated CTCs from healthy blood cells via CD45 depletion (RosetteSep™ system) as a pre-enrichment step, followed by CTC identification with three CSM markers: EpCAM, pan-CK, and VIM. EpCAM and pan-CK recognized antigens/epitopes present on epithelial CTCs, whereas VIM captured CTCs undergoing EMT. They invented a simple, fast, sensitive, and higher recovery technique to detect both epithelial and mesenchymal CTCs that could be complemented, when needed, by other in-depth analyses [112]. In 2020, Hamid et al. claimed that prior to the CD45-based subtraction of hematogenous cells, CTC enrichment with both EpCAM and CK markers enabled the authors to relate CRC staging with CTC morphological and phenotype features [102]. 

On the other hand, Bahnassy and the coresearchers combined multiple enrichment methods to identify CTCs. Briefly, CD45-based negative enrichment was utilized to subtract nontarget cells. The validation of CTC was performed by a combination of CellSearch, cytomorphology, flow cytometry (FCM), and real transcriptase quantitative PCR (RT-qPCR) with multiple markers including cytokeratin (CK3, CK19), mucin 1 (MUC1), CD44, CD133, and aldehyde dehydrogenase 1 (ALDH1). In this comparative study, they confirmed the superiority of multiplexing several different techniques (positive detection rate: 68.3%) over a single CTC enrichment strategy (positive detection rate: 54% (CellSearch); 50.8% (FCM)). Interestingly, CTCs were identified as novel therapeutic targets for nonmetastatic CRC [98]. To sum up, despite numerous benefits over traditional single CSM-based CRC detection, multiplexing on CTC enrichment remains very limited [113].

## 7. Circulating Cancer Stem Cells Are a Rare CTC Subtype

Until today, the mechanisms between CTCs and circulating cancer stem cells (CCSCs) remain unclear [114]. There are, however, increasing evidence revealing the existence of cellular heterogeneity within CTCs [40], and that a presumably small subset of them harbor cancer stem cell characteristics due to their ability to survive in the blood, and resist chemotherapy and progression into metastatic lesions [115,116,117]. For instance, in 2017, Grillet et al. demonstrated that CTCs from CRC patients exhibited cancer stem cell hallmarks when culturing ex vivo [115]. Furthermore, CTCs and CCSCs show different functional states of the same pathogenically relevant cancer cell subpopulations [118,119]. Thus, the identification of drug-resistant CTCs in the bloodstream would, at least theoretically, provide a unifying hypothesis, where CCSCs might be a rare CTC subtype [120].

As CCSCs are likely to represent small subsets of CTCs, the traditional CSM-based CTC enrichment method could be applied in identifying CCSCs. In 2020, a group of researchers in Italy shed insights toward the possibility of using the anti-human CD44v6 antibody to detect the CCSC subpopulation from patients-derived CTCs. The CD44v6 isoform was selected for several reasons: (1) involvement in cancer cell migration and invasion; (2) functional biomarker of stemness and therapeutic target in CRC [121]; (3) presence in all CRC stem cells (capable of metastatic tumors generation) [122]; and (4) the highly expressed CD44v6 protein on CTCs with functional attributes of CCSCs [123]. Briefly, a single CSM EpCAM was selected to positively enrich CTCs from peripheral blood. The isolated CTCs were then verified with CK8, CK18, CK19, CD44v6, and CD45. Their research demonstrated that the enumeration of CD44v6-positive CTC/CCSC obtained from mCRC patients could be used to early detect intrinsic drug resistance, as well as predict the first-line treatment failure [124]. In short, the CSM-based enrichment technique showed potential in isolating CCSCs for CRC screening and tumor response prediction in mCRC patients. Table 1 summarizes the common CTC enrichment techniques categorized by specific CSMs for CRC screening.

## 8. Importance of Enrichment Technique over Selection of CTC Analysis and Characterization in the CRC Screening Stage

Ideally, CTC enrichment would provide pure CTC for enumeration, as well as downstream analysis, and reflect the total CTC status in CRC patients. As one of the commonly used techniques, biophysical property-based enrichment systems are capable of isolating CTCs via the specific selection of size, density, or deformability. Although a wider subset of CTCs could be enriched due to its independence on the CSM, the recovery efficiency is limited due to the buildup of filtration resistance, formation of CTC aggregates, and membrane clogging [125,126]. 

On the other hand, functional and nucleic acid-based CTC enrichment are widely used alternatives to immunoaffinity-based systems. These techniques identify specific tumor markers to confirm the presence of CTCs indirectly [127,128]. For instance, the immunocytochemistry of CTCs permits CTC morphological analysis and labeling of specific ligands [129,130], whereas qRT-PCR allows the detection of specific biomarkers with high sensitivity [131]. Post-functional assays, where enriched CTCs are cultured in 2D or 3D models, also enable the evaluation of migration and invasion abilities [132,133]. The advancement in NGS technologies even granted the possibility of dissecting CTC at the single-cell level [134]. Nonetheless, they lack specificity due to the potential to capture noncancerous cells to generate false-positive signals, thus decreasing the overall accuracy. The lack of standardized sampling and pre-enrichment methods might also result in the significant loss of CTCs. Other fatal clinical implications include (1) high contamination risks with hematopoietic cells/white blood cells; (2) denaturation/lysis of CTCs; (3) lack of certain CTC subpopulations due to unspecific markers during enrichment; and (4) the isolated CTCs might not reflect the actual CTC status of patients, resulting in bias or false results. In our opinion, these limitations could be overcome if functional and nucleic acid-based CTC enrichment techniques are incorporated as downstream analysis for CTC analysis and characterization. Thus, we believe that the multi-CSM-based CTC isolation system is a promising enrichment strategy due to its higher CTC capture efficiency and higher specificity. Moreover, the enumeration of enriched CTCs, alone, has been proven to be adequate for CRC screening, prognosis, and disease progressing monitoring [135]. Table 2 summarizes advantages and disadvantages of single and multi-CSM based enrichment over other CTC enrichment methods.

## 9. Stigma on Circulating Tumor Markers in Blood 

Fundamentally, CSM-based CTC enrichment techniques rely on screening the peripheral blood for CTC collection. As the antigens’ expression of CTCs and their specific phenotypic characteristics affect the CRC progression and patient survival, CSMs have become the main focus for CTC enrichment. However, there is an ongoing debate between CSM and circulating tumor markers in the blood, where the latter could potentially detect CTCs without the isolation from whole blood [136]. The concept of a circulating tumor marker applies to a chemical product originated from a CRC cell (including CTC), such that its concentration in the blood represents a quantifiable assessment of the tumor burden at a specific time [137].

Some examples for the blood-based circulating tumor marker include proteins [46,138,139,140]; low-molecular-weight metabolites (volatile organic compounds) [141]; DNA (including methylation markers) [142,143,144,145,146,147]; and RNA (messenger RNA, non-coding RNA, and microRNA) [148,149,150,151]. These blood biomarkers are also capable of the detection of tumor-specific mutations associated with the response to targeted therapy. Nevertheless, the analytical specificity might be limited as they could potentially be released from necrotic or apoptotic cells, as well as active secretions by intact cells, of tumor origin or/and from nontumor origins, including hematopoietic, immune, and blood stromal cells [152,153,154,155]. 

In 2020, Liu et al. discovered three identical mutations in both cell-free DNA (cfDNA) and CTCs, seven mutations found only in the cfDNA, and one exon 19 deletion in the CTCs from 11 EGFR-mutated cancer patients. Their results proved the supremacy of the combination of cfDNA and CTCs over either test alone. Interestingly, in the third subsequent blood draw, the previous exon 19 deletion could not be detected. The reduction in CTC concentration due to chemotherapy/cell apoptosis resulted in DNA from CTCs being released into the blood, resulting in the better performance of cfDNA than CTCs. In other words, new mutations would be first detected in the CTCs, while cfDNA would provide a snapshot of dying cancer cells instead [156]. Consequently, instead of replacing CSM-dependent CTC enrichment, blood-based circulating tumor markers represent complementary predictive cancer biomarkers, as well as real-time CRC monitoring in clinical practice [157]. 

## 10. Challenges in Routine Implementation of CTC-Specific CSM-dependent CRC Detection 

Despite the discovery of numerous CTC-specific CSMs, the main limitation that hampers existing CTC detection technologies is still the a priori knowledge of the exact protein composition on the CTCs surfaces, and the lack of a universal marker(s) to address the heterogeneity of CTCs in CRC [125,158,159]. The current gold-standard technique for CTC detection, the microscopic cell imaging, also presents many drawbacks such as the low number of markers, inability to analyze multiple markers simultaneously in routine use, long turnaround time (incompatible with the urgent need for delivery of treatment), and the requirements for specific laboratory instruments and professional expertise (pathologists) for data analysis [160,161]. Furthermore, the lack of large-population follow-up cohort studies increases the difficulties of translating current CSM-based CTC detection methods into the clinical setting for CRC screening, diagnosis, prognosis, real-time monitoring, and therapeutic response [50,51,52,53,54]. Other reasons include (i) the vast number of methods described for potential CTC detection (including the pre-analytical, analytical, and post-analytical phases), without a consensus on the ideal/standardized technical approach; (ii) difficulty in controlling the pre-analytical phase to obtain robust and reproducible results; and (iii) the high cost of the currently available techniques [162,163]. 

## 11. Conclusions

Cell surface markers/antigens on CTCs are crucial markers for the diagnosis and prognosis of metastatic and nonmetastatic CRC. Despite the potential scientific and medical usefulness of current CTC enrichment technologies, adopting them into the clinical setting will demand laborious studies into their analytical validity, clinical validity, and clinical utility. Therefore, the standardization of all procedures should be emphasized. A multi-marker-based system is believed to permit the enrichment of a wider subset of CTCs, including phenotypes of epithelial, mesenchymal, and those transitioned in between. Nevertheless, additional large-scale studies in high-risk groups and the further understanding of their biology and significance could enhance CTCs’ utility as a blood-based biomarker [163]. Finally, a real gap exists between the genuine attraction of obtaining a large number of publications in this domain and its application into routine clinical practice.

## Figures and Tables

**Table 1:** CTC-specific cell surface marker (CSM)-based enrichment techniques for CRC screening.

Feature	Surface Marker	CTC Identification Marker	CTC Enrichment Technique	Principle/Technology	Pros and Cons	Positive Detection Rate	Study	Clinical Utility	Ref.
Single specific CSM	EpCAM	CK8, CK18, CK19, HER2, CD45, DAPI, Hoechst	CellSearch® system (Veridex)	Positive enrichment; ferromagnetic beads labeled with EpCAM-antibodies to capture CTCs; identification of CTCs via staining with CK8, CK18, CK19, and HER2; CD45 marker to exclude hematogenous cells; DAPI/Hoechst as marker to identify intact CTCs; CTC enumeration via CellTrack Analysis II	FDA-approved for advanced CRC; loss of EpCAM-negative CTCs; lack of EMT detection; no further downstream analysis	88.9% (32/36 patients)	Prospective, multicenter, nonrandomized trial (NCT00994864)	Prognosis and prediction of CRC	[74]
	EpCAM	CK20, CD45, DAPI	CellMax platform (CellMax Life)	Positive enrichment; microfluidics chip technology platform; EpCAM-coated SLB to capture CTCs; identification of CTCs via staining with CK20; CTCs enumeration via AI-based automated CellReviewer	Loss of EpCAM-negative CTCs; lack of EMT detection; CTCs intact for downstream analysis	43.8% (14/32 patients)	Cohort study	CRC screening	[82]
	EpCAM	CK20, CD45, DAPI	CellMax platform (CellMax Life)	Positive enrichment; CTCs enrichment using EpCAM antibody and stained using CK20 for confirmation; CTC enumeration via an algorithm in CellFinder software	Loss of EpCAM-negative CTCs; lack of EMT detection	94.5% (307/325 patients)	Bioanalytical assay development and validation study	CRC screening	[30]
	EpCAM	CK8, CK18, CD45, DAPI	Polymeric CTC chip	Positive enrichment; polymeric microfluidic chip coated with EpCAM for CTC detection; staining with CK8 and CK18 for CTC validation; manual CTC enumeration under an inverted fluorescence microscope	Loss of EpCAM-negative CTCs; easily blocked chips; lack of EMT detection	92.3% (12/13 patients)	Comparative, longitudinal study	CRC screening; CRC progression monitoring and treatment effects	[83]
	EpCAM	CK-7, CK-8, CK-18, CK-19, CD45, DAPI, Hoechst	IsoFlux (Fluxion Biosciences)	Positive enrichment; EpCAM- based magnetic separation by flow cytometry to capture CTCs; identification of CTC via CK-7, CK-8, CK-18, and CK-19 markers; CTC enumeration via IsoFlux	Loss of EpCAM-negative CTCs; detection of CTC and MSI status in the peri-operative colorectal surgery setting; lack of EMT detection; no further downstream analysis	95% (19/20 patients)	Cross-sectional study	CRC screening; CRC progression monitoring and treatment effects	[84]
	EpCAM or KRAS	CK20, CD45, DAPI	MACS/ microemulsion method	Positive enrichment; EpCAM or KRAS-coated lipid bilayer encapsulated superparamagnetic Fe_3_O_4_ nanoparticles balls to capture CTCs with EpCAM expression or KRAS mutation; identification of CTC via CK20 and CD45; KRAS detection via PCR	Detection of CTCs with KRAS mutation; intact CTCs for PCR to validate KRAS mutation	100% (KRAS) (55/55 patients); 54.5% (EpCAM) (30/55 patients)	Comparative study	KRAS mutation detection; provide diagnosis and treatment of KRAS CRC	[111]
	EpCAM	pan-CK, CD45, DAPI, Hoechst EpCAM, pan-CK, CD45, DAPI, Hoechst	CellSearch® system (Veridex) GILUPI CellCollector	Positive enrichment; ferromagnetic beads labeled with EpCAM antibodies; identification of CTCs via staining with pan-CK Positive enrichment; novel in vivo CTC detection device with EpCAM, followed by pan-CK/EpCAM (double-staining) for verification	FDA-approved; loss of EpCAM-negative CTCs; lack of EMT detection Loss of EpCAM-negative CTCs; lack of EMT detection	31.3% (25/80 patients) 41.3% (33/80 patients)	Prospective, single center, investigator-blinded side-by-side comparative study	Prediction of CRC (overall survival based on staging) CRC screening	[96]
	EpCAM	CK8, CK18, CK19, CD44v6, CD45	CellSearch® CXC kit (Menarini Silicon Biosystems)	Positive enrichment; ferromagnetic beads labeled with EpCAM-antibodies to capture CTCs; identification of CCSCs via CD44v6 expression	Identification of CTCs with functional attributes of CCSCs via CD44v6 expression	62.5% (25/40 patients)	Bioanalytical assay development study	mCRC screening; prediction of first-line treatment failure and tumor response in mCRC patients	[124]
	CD45	DAPI	Cyttel method	Negative enrichment; CD45-based immunomagnetic system to remove hematogenous cells; CTC identification via imFISH of chromosomes 8 and 17 H1 fluorescent probes, together with DAPI staining	Loss of significant cells; low purity	58.7% (71/121 patients)	Retrospective study	CRC screening; prediction of survival outcome	[99]
Multi-CSM	CD2, CD16, CD19, CD36, CD38, CD45, CD66b, and glycophorin A	CK19, VEGF	RosetteSep™ System (StemCell Technologies)	Positive enrichment; immunodensity procedure; RosetteSep™ tetrameric antibody complexes crosslink unwanted hematogenous cells; isolation of CTCs via density gradient centrifugation, followed by EPISPOT assay where specific secreted proteins were captured by antibody-coated membrane; counting of immunospots (one immunospot corresponded to the protein fingerprint of one viable cell)	Many CTCs harvested; detection of viable CTCs at the single-cell resolution; utilization of CTC-secreted proteins for enrichment; possible for protein characterization	-	Bioanalytical assay development study	CRC screening; possible protein characterization	[100]
Post multi-CSM	CD45	pan-CK, EPCAM, VIM, DAPI	RosetteSep™ System (StemCell Technologies)	Pre-negative CD45 enrichment to exclude hematogenous cells; secondary enrichment with EpCAM and CK. Pan-CK, EPCAM, and VIM; CTC enumeration via FCM	Simple, fast, sensitive, and higher recovery to detect potential CTCs (with EMT); CTCs intact for downstream analysis	46.7% (7/15 patients)	Case control	CRC screening	[112]
	CD45	EpCAM, CK	EasySep™ (StemCell Technologies)	Pre-negative magnetic CD45 enrichment to exclude hematogenous cells; secondary enrichment with EpCAM and CK; manual CTC enumeration	Little clinical relevance of CTC number to CRC staging; CTC morphology and phenotype closely related to CRC stages	72% (41/57 patients)	Case control	CRC screening	[102]
	CD45	CK18, CEP8, DAPI	SE-iFISH (Cytelligen)	Pre-negative CD45 enrichment to exclude hematogenous cells; secondary enrichment with EpCAM and CK with anti-CK18 and anti-CEP8	Identification of CTC optimal detection time (after at least 7 postoperative days)	85% (17/20 patients)	Cross-sectional cohort study	CRC screening and postoperative monitoring	[101]
Combined approach	CD45	CK3 CK3, CK19, MUC1, CD44, CD133, ALDH1,	CellSearch® system (Veridex) CellSearch + cytomorphology + FACS + RT-qPCR	Negative enrichment; CTCs were isolated via CD45+ cells depletion kit and further enriched with anti-CK3-labeled magnetic beads. Combination of several CTC-negative enrichment techniques	CTCs as novel therapeutic targets for nonmetastatic CRC Improved sensitivity and specificity	54% (34/63 patients) 68.3% (34/43 patients)	Comparative study	CRC screening	[98]

ALDH1 = aldehyde dehydrogenase 1, CCSC = circulating cancer stem cell, CD = cluster of differentiation, CEP8 = centromeric probe for chromosome 8, CRC = colorectal cancer, CTC = circulating tumor cells, CK = cytokeratin, DAPI = 4′,6-diamidino-2-phenylindole, EMT = epithelial–mesenchymal transition, EpCAM = epithelial cell adhesion molecule, FACS = fluorescent-activated cell sorting, FCM = flow cytometry, MACS = magnetic-activated cell sorting, MUC1 = mucin 1, RT-qPCR = reverse transcriptase quantitative polymerase chain reaction, SE-iFISH = subtraction enrichment and immunostaining-fluorescence in situ hybridization, VIM = vimentin.

**Table 2:** Comparisons of most commonly used CTC enrichment methods.

CTC Enrichment Technique	Advantages	Disadvantages
Biophysical isolation (size/microfiltration; density gradient centrifugation)	Quick and simple way to isolate CTCs; Label-free CTC isolation; Rapid processing of large volumes; Applicable to all types of cancers; Inexpensive; Harvest a wider subsets of CTCs	Poor sensitivity due to the loss of some CTCs during migration or formation of CTC aggregates or membrane clogging; Low specificity; Stringent sampling procedure (blood samples collected must be processed immediately and required pre-enrichment step); High contamination risks with hematopoietic cells; Limited due to the heterogeneity in the size and density of CTCs
Single CSM-based system (a) Positive enrichment	Clinically validated (FDA-approved system); Robust and reproducible; Specific to certain CTC subpopulation depending on the selected marker (epithelial or mesenchymal trait); Advancement in microfluidics technology allows intact cells for downstream analysis	Significant loss of certain CTC subpopulations if a single CSM is used for positive enrichment; Inability to address several parameters (e.g., EMT and mutations) due to the use of single CSM; Inability to address totality of CTCs; Lack of cancer-specific CSMs
(b) Negative enrichment	Capable of harvesting all types of CTCs if negative enrichment was applied; High CTC viability; No bias based on CSMs; More competent for the discovery of cellular and transcriptomic cancer biomarkers of cancer and downstream analyses such as genetic assays, CTC culture, and xenografts	Low purity and specificity due to the loss of CTCs, especially during negative enrichment; Uncertainty in the accuracy to identify a patient’s CTC status
Multi-CSM-based system	Higher yield than single CSM-based enrichment systems; High CTC capture efficiency (CTCs of different origins were captured by covering epithelial, mesenchymal, and stem cell markers); Increased analytic sensitivity and specificity than single CSM-based system; Capable of addressing the totality of CTCs; Advancement in microfluidics technology allows intact cells for downstream analysis	Lack of specific combinatorial list of CSMs; Unstandardized protocols when multiple markers are used (e.g., marker concentrations and incubation time); Lack of automated procedures; Limited studies using multiple CSMs for pre-CTC enrichment step
CCSC-targeted CTC enrichment	Identification of CTCs with cancer stem cell characteristics; More selection of CSMs (including Lrg5, DCLK1, and ANXA2); More specific for mCRC screening; Drug resistance identification	Low population in CTCs; Limited studies
Nucleic acid-based or functional-based enrichment system/post-CTC analysis and characterization (e.g., immunocytochemistry, qRT-PCR, ddPCR, EPISPOT, NGS, and functional assays)	Detection of viable CTCs; Evaluation of CTC migration and invasion abilities; Ability to address cellular heterogeneity; Capable of CTC characterization; Capable of single cell resolution analysis; Permit CTC morphology analysis; Detection of specific markers not limited to the surface of CTCs	Unstandardized sampling method resulting in significant loss of CTCs and high contamination risks with white blood cells; Unspecific markers for enrichment resulting in the loss of certain CTC subpopulations; Isolated CTCs might not reflect the actual CTC status of patients; Bias or false negative results due to loss of CTCs during the enrichment step; No possibility to recover CTCs

CCSC = circulating cancer stem cell, CSM = cell surface marker, CTC = circulating tumor cell, CRC = colorectal cancer, ddPCR = digital droplet, EMT = epithelial-to-mesenchymal transition, polymerase chain reaction, mCRC = metastatic CRC, NGS = next-generation sequencing, qRT-PCR = quantitative reverse transcriptase polymerase chain reaction.

## Abbreviations

AJCC	American Joint Committee on Cancer
CCSCCD	Circulating cancer stem cellsCluster of differentiation
cfDNA	Cell-free DNA
CK	Cytokeratin
CRC	Colorectal cancer
CSM	Cell surface marker
CTC	Circulating tumor cell
DAPI	4′,6-diamidino-2-phenylindole
EMT	Epithelial–mesenchymal transition
EpCAM	Epithelial cell adhesion molecule
FACS	Fluorescent-activated cell sorting
FCM	Flow cytometry
FDA	Food and Drug Administration
KRAS	Kirsten rat sarcoma viral oncogene
MACS	Magnetic-activated cell sorting
mCRC	Metastatic CRC
pan-CK	Pan-cytokeratin
RT-qPCR	Reverse-transcriptase quantitative polymerase chain reaction
SE-iFISH	Subtraction enrichment and immunostaining-fluorescence in situ hybridization
VIM	vimentin
